# Transfer Effects of Integrated Adaptive Working Memory Training in School-Age Children: Improvements in Inhibitory Control and Asymmetric Gains in Mathematical Abilities

**DOI:** 10.3390/jintelligence14070140

**Published:** 2026-07-05

**Authors:** Yuan Tao, Hao Li, Haisheng Fu, Xiaotong Zhang, Xiao Yu, Andy Yu

**Affiliations:** 1 School of Psychology, Beijing Sport University, Beijing 100084, China; taoyuan@bjfu.edu.cn; 2 School of Soil and Water Conservation, Beijing Forestry University, Beijing 100083, China; 3 The Hospital for Sick Children, Toronto, ON M5G 1X8, Canada; hao.li@sickkids.ca; 4 Department of Medical Biophysics, University of Toronto, Toronto, ON M5G 1L7, Canada; 5 MeowSprout Inc., Coquitlam, BC V3E 0T5, Canada; haisheng.fu@ubc.ca; 6 Department of Electrical and Computer Engineering, University of British Columbia, Vancouver, BC V6T 1Z4, Canada; 7 Department of Psychology, School of Humanities and Social Sciences, Beijing Forestry University, Beijing 100083, China; hope511@bjfu.edu.cn (X.Z.); yuxiao_psy@bjfu.edu.cn (X.Y.)

**Keywords:** approximate calculation, cognitive plasticity, exact calculation, inhibitory control, school-age children, working memory training

## Abstract

The efficacy of working memory training (WMT) in facilitating academic achievement remains heavily debated. To resolve this efficacy paradox, the current study implemented an integrated adaptive WMT (IA-WMT) paradigm to investigate near transfer to inhibitory control (IC) and far transfer to mathematical abilities in school-age children. We assigned 89 typically developing children (*M_age_* = 109.08 ± 3.90 months; 43 girls) to an IA-WMT group (29 students), an active control group (30 students), or a passive control group (30 students). Pretest and posttest assessments measured core working memory capacity, IC, exact calculation (EC), and approximate calculation (AC). Results revealed that six sessions of IA-WMT significantly enhanced visual–spatial and verbal working memory. Furthermore, the training induced potential near transfer to IC and asymmetric far transfer to EC, while yielding no specific gains for AC. This functional dissociation provides valuable alignment with the dual-system model of mathematical cognition. The findings suggest that far transfer may not operate as a generalized spillover effect but appears to be governed by the degree of functional overlap between trained executive processes and inherent task demands. Ultimately, organically integrated cognitive training represents a promising targeted intervention tool to facilitate cognitive and academic development during early school years.

## 1. Introduction

Working memory (WM) constitutes a core component of human cognition and is intimately linked to daily functioning and quality of life (H.-H. Li et al., 2025; G. Li et al., 2026). Defined as the cognitive faculty responsible for the concurrent maintenance and manipulation of information (Baddeley, 1992, 2012), WM serves as the primary cognitive engine that drives both inhibitory control (IC) and mathematical proficiency in school-age children (Chan et al., 2024; Constantinidis & Klingberg, 2016; Hitchcock & Westwell, 2017). Within the framework of the multicomponent model, WM is conceptualized as an active workspace managed by the central executive, which is responsible for maintaining target representations and coordinating complex mental operations (Baddeley, 1992, 2012). Theoretically, the “capacity” of this system is not a fixed structural limit but a manifestation of the efficiency of underlying executive processes; high-capacity individuals excel at prioritizing task-relevant information while effectively inhibiting interference (Engle & Kane, 2004).

Despite its importance, contemporary research has predominantly operationalized working memory training (WMT) through a narrow focus on the “updating” function (Bai et al., 2026; Miyake et al., 2000; Pappa et al., 2020). While not denying the role of other executive processes, this singular focus often compromises training quality and intervention depth (Aksayli et al., 2019; Bergman Nutley & Söderqvist, 2017; Willoughby et al., 2023), leading to a phenomenon characterized as an “efficacy paradox”—a state of inconsistent findings regarding near and far transfer effects (Kassai et al., 2019; Pahor et al., 2022). This empirical fragmentation suggests that the latent capacity for cognitive reorganization during these critical school years might be better understood by exploring intervention depth rather than assuming a lack of behavioral malleability.

To resolve these long-standing empirical discrepancies, current methodological frameworks advocate for a transition toward integrated task paradigms implemented through rigorous experimental designs (Almaatouq et al., 2024; Carr et al., 2023; Redick et al., 2015). By deploying the integrated adaptive working memory training (IA-WMT) alongside a robust active control group, the present study seeks to overcome the limitations of isolated drills and provide a more nuanced investigation into near transfer to IC and far transfer to mathematical abilities. Specifically, we address critical measurement gaps by using reaction-time-based efficiency metrics for IC, while simultaneously delineating the differential impact of training on exact calculation (EC) versus approximate calculation (AC).

### 1.1. Integrated Adaptive Working Memory Training for School-Age Children

Research has established that computerized WMT yields significant and robust gains in cognitive performance among school-age children (Kassai et al., 2019; Sala & Gobet, 2017). Meta-analytic evidence confirms that these interventions produce stable practice effects across diverse populations, effectively enhancing the functional capacity of the WM architecture (Kassai et al., 2019; Melby-Lervåg et al., 2016). However, while the basic efficacy of WMT is well-documented, a critical gap remains regarding the integration of training components. Traditional interventions often rely on isolated task drills that fail to capture the synergy between distinct executive processes (Carr et al., 2023; Redick et al., 2015). To bridge this gap, the present study developed an IA-WMT paradigm, designed as a unified cognitive workspace rather than a collection of disparate tasks, requiring children to simultaneously manage diverse executive demands. The “Integrated” nature of IA-WMT is operationalized across functional, formal, and strategic dimensions. Functionally, it merges updating and IC tasks to target the overarching central executive rather than isolated sub-functions (Miyake et al., 2000; Saylik et al., 2022; Scharinger et al., 2015). Formally, it synchronizes core cognitive tasks with gamification and thematic scenarios to enhance ecological validity (Cicerone et al., 2019; Hamari et al., 2014; Song et al., 2023). Strategically, IA-WMT creates a robust synergy between adaptivity-driven neuroplasticity (Barbazzeni et al., 2023; Jones et al., 2022) and strategy-based instruction, providing efficient mnemonic shortcuts (Assecondi et al., 2021; Meneghetti et al., 2018). This multifaceted approach aims to provide children with the opportunity to not only expand their structural processing limits but also refine their cognitive toolkits to manage information more efficiently (Ben Izhak & Lavidor, 2023; Lövdén et al., 2012).

Furthermore, the structural integrity of IA-WMT is fortified by dynamic adaptive mechanisms, scenario embedding and gamification. While adaptive algorithms provide the necessary challenge by maintaining the task difficulty at the individual’s upper capacity threshold (Klingberg, 2010; Sweller, 2020), gamification and scenario embedding provide the scaffolding necessary to sustain high training dosages and mitigate cognitive fatigue (Y. Liu et al., 2026; Wang et al., 2025). Therefore, the IA-WMT seeks to bridge the gap between laboratory-based efficacy and real-world cognitive reorganization by holistically addressing the complexity of the developing executive system.

### 1.2. Integrated Adaptive Working Memory Training Induces Multidimensional Transfer Effects in School-Age Children

#### 1.2.1. Organically Integrated Cognitive Demands Dictate the Efficacy of near Transfer to Inhibitory Control

The near transfer from WMT to IC is fundamentally rooted in the optimization of the central executive, which serves as the foundational cognitive platform for coordinating strategic modes (Baddeley, 2012; Kattner, 2021; Miyake et al., 2000). Within this framework, IC functions as a cognitive gatekeeper, maintaining goal representations by filtering irrelevant distractions (Chiew & Braver, 2017; Dempster, 1992). Crucially, total working memory capacity represents the outward manifestation of underlying executive efficiency, where the expansion of functional capacity is achieved by enhancing the ability of the gatekeeper to prioritize task-relevant information (Baddeley, 2012; Engle & Kane, 2004). However, existing WMT paradigms targeting isolated updating functions often fail to challenge this maintenance process under high-interference conditions (Guye & von Bastian, 2017; Shipstead et al., 2012). By organically integrating both updating and inhibitory demands, IA-WMT challenges the central executive under high-interference conditions, allowing us to test whether the subsequent improvement of IC occurs as a consequential transfer effect.

Theoretical links between WM updating and IC are increasingly supported by neurobiological models and reconciled with the inconsistent empirical outcomes in intervention research (Berger et al., 2025; Diamond & Ling, 2016; Nichols et al., 2021). Supported by longitudinal evidence of white matter maturation, it is proposed that only when individuals possess sufficient functional capacity to stably maintain task goals can the brain transition from stimulus-driven reactive control to pre-emptive proactive strategies, thereby bypassing the need for late-stage corrections and manifesting as improved efficiency in reaction-time metrics (Berkman et al., 2014; Delalande et al., 2020; Munakata et al., 2011). Furthermore, empirical evidence for near transfer remains inconsistent, as traditional single-component training is often too narrow to trigger broad cognitive reorganization (Basak et al., 2020; Kassai et al., 2019), while standard multicomponent programs risk weakening intervention effects due to the dispersion of cognitive resources across diverse tasks (Ludyga et al., 2023). In school-age samples, traditional accuracy-based metrics often fail to capture nuanced progress in processing efficiency due to prevalent ceiling effects, making reaction-time-based analyses essential for reflecting the shift toward proactive control (Diamond & Ling, 2016; Willoughby et al., 2023). Additionally, compared to traditional interference indices that frequently suffer from low reliability in developmental populations (Draheim et al., 2019), utilizing the mean reaction time provides a more robust indicator of the global shift toward proactive control. The current study seeks to bridge these gaps by utilizing the IA-WMT framework to circumvent measurement limitations and provide further empirical evidence regarding the malleability of inhibitory functions during the school years.

#### 1.2.2. Differential Cognitive Resource Demands Dictate the Scope of Far Transfer to Mathematical Abilities

Far transfer from WMT to mathematical abilities may be fundamentally rooted in the expansion of core cognitive capacity, which provides critical scaffolding for complex computational processes (Bergman Nutley & Söderqvist, 2017; L. Fuchs et al., 2020; L. S. Fuchs et al., 2022). WM directly influences mathematical efficiency by providing the necessary cognitive resources for the online storage of intermediate results and the execution of strategic problem-solving procedures (Beilock & DeCaro, 2007; Cragg et al., 2017; Titz & Karbach, 2014). While several investigations suggest that adaptive, training-induced capacity augmentation can translate into academic gains (Jones et al., 2020; L’Hotta et al., 2025; Nelwan et al., 2018), the efficacy of this far transfer is not universal, as the recruitment of cognitive resources varies significantly across different mathematical sub-domains. According to the dual-system model of mathematical cognition, numerical processing is governed by two functionally dissociable systems (Dehaene et al., 1999; Stanescu-Cosson et al., 2000). Neuroimaging evidence indicates that EC relies predominantly on language-driven algorithmic processing supported by the left inferior frontal gyrus and the left angular gyrus, which necessitates high levels of real-time WM maintenance and manipulation. Conversely, AC primarily activates the approximate number system associated with the bilateral intraparietal sulcus, favoring non-verbal intuitive representations that impose significantly lower demands on precise working memory capacity (Ganor-Stern, 2018; Kucian et al., 2008; F. Liu, 2013). Therefore, the spillover effect of cognitive training likely manifests through specific cognitive pathways, suggesting that the degree of transfer is fundamentally constrained by the inherent processing demands of the academic task.

Despite the robust theoretical foundation, empirical conclusions remain markedly divergent (Aksayli et al., 2019; Hitchcock & Westwell, 2017; Sala & Gobet, 2020). Beyond the constraints of single-task designs or insufficient stimulation intensity, which are often inadequate to induce the profound cognitive reorganization required for academic transfer, inconsistencies stem primarily from significant cross-experimental variations in intervention paradigms and the selection of assessment tools (Bergman Nutley & Söderqvist, 2017; Pergher et al., 2020; Titz & Karbach, 2014). Targeting the critical transition period of development of mathematical abilities during the school years (Jordan et al., 2003; Vogel & De Smedt, 2021), the present study utilizes the dual-system model as an interpretive framework to examine these mixed findings. By deconstructing mathematical abilities into EC and AC within an ecologically valid paradigm, this research tests whether training-induced transfer effects align with the specific cognitive processing demands of these mathematical sub-domains.

### 1.3. The Current Study

Drawing on the framework of Wilkinson et al. (2020), this research categorizes cognitive gains into three distinct levels: training effects within the targeted domain, near transfer to related executive functions like IC, and far transfer to non-executive domains such as mathematical abilities. Regarding near-transfer effects, the investigation of IC is essential during the school years, as this cognitive function serves as the cornerstone for behavioral regulation and successful adaptation to the formal school environment (Gagne et al., 2025; Joyce et al., 2016). Despite its significance, whether WMT can produce stable and replicable gains in IC remains a point of significant contention in the developmental literature, with meta-analytic evidence often showing mixed or inconsistent results (Basak et al., 2020; Kassai et al., 2019). Similarly, mathematical abilities act as robust predictors of subsequent academic success and long-term development (Geary, 2000; Martin, 2018; Vogel & De Smedt, 2021). Notwithstanding this critical role, the capacity for WMT to induce meaningful gains in mathematical abilities remains a subject of considerable debate within the developmental literature (L’Hotta et al., 2025; Sala & Gobet, 2020). Given that the school years represent a critical window for the concurrent development of WM, IC, and mathematical abilities (Hartung et al., 2020; Hawes et al., 2019), the current study situates WMT within an integrated framework to address the existing methodological fragmentation. Adhering to established methodological standards that emphasize the use of multicomponent paradigms and active control groups for catalyzing school-age children’s cognitive plasticity (Melby-Lervåg et al., 2016; Redick et al., 2015), this research investigates whether the IA-WMT can facilitate detectable near and far transfer beyond the practiced tasks. Crucially, the study utilizes Huarongdao sliding block puzzles for the active control condition to strategically account for nonspecific motivational effects and baseline visuospatial sequential problem-solving. By matching these elements, the design aims to isolate the cognitive plasticity potentially driven by the high-intensity, cumulative adaptive WM updating demands inherent to the IA-WMT. Specifically, the study pursues three primary objectives. First, we evaluate the effectiveness of IA-WMT in enhancing core working memory capacity across verbal and visual–spatial domains, hypothesizing that the integrated, adaptive nature of the intervention will yield superior gains compared to both control conditions. Second, we investigate the near transfer to IC using reaction-time-based efficiency metrics, positing that IA-WMT will promote the shift toward more efficient proactive control strategies. Finally, we delineate far transfer to mathematical abilities by distinguishing between EC and AC. We hypothesize that training-induced capacity expansion will selectively enhance EC, which relies on high-load algorithmic manipulation, while exerting minimal impact on AC, which is predominantly driven by intuitive representations.

## 2. Materials and Methods

### 2.1. Participants

Prior to recruitment, G*Power 3.1 (Heinrich-Heine-Universität Düsseldorf, Düsseldorf, Germany) estimated a minimum of 42 participants for a 3 × 2 mixed-design analysis of variance (ANOVA). This specific calculation assumed an effect size of 0.25 alongside an alpha level of 0.05 and a statistical power of 0.80 (Faul et al., 2009). We subsequently raised the final enrollment target to 30 individuals per experimental condition. This deliberate expansion guarantees methodological robustness and prevents the effect size inflation frequently observed in cognitive training research (Melby-Lervåg et al., 2016). We recruited a total of 90 children (*M_age_* = 109.07 ± 3.88 months; 43 girls) from an elementary educational facility located in Shandong, China through a convenience sampling approach. Participants were allocated to the IA-WMT, active, or passive control group in a 1:1:1 ratio. This process utilized a computer-generated randomization sequence managed by a researcher blinded to the study’s specific hypotheses and subsequent assessment procedures.

During the intervention, one participant from the IA-WMT group withdrew due to scheduling conflicts. Consequently, the final sample consisted of 89 children (*M_age_* = 109.08 ± 3.90 months; 43 girls): 29 in the IA-WMT group (*M_age_* = 108.84 ± 3.10 months; 15 girls), 30 in the active control group (*M_age_* = 110.04 ± 5.53 months; 13 girls), and 30 in the passive control group (*M_age_* = 108.36 ± 2.20 months; 15 girls). Statistical comparisons confirmed that the three groups were well-matched at baseline in terms of chronological age, *F*(2,86) = 1.37, *p* = .26, and gender distribution, *χ*^2^(2) = 0.47, *p* = .79. Every participant possessed regular or medically corrected sensory acuity for both sight and sound and had no prior history of participation in cognitive training programs. The legal representatives for every child participating in the research granted their formal permission through signed consent documentation. Upon completion of the full experimental procedure, children received age-appropriate incentives to acknowledge their contribution to the research. The institutional review board at the university granted formal ethical clearance for this investigation.

### 2.2. Materials

#### 2.2.1. Corsi Block Task

**Figure 1:**
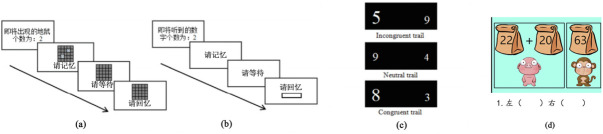
Visual configurations of the assessment task modules. Note. The panels illustrate example trials and layouts for each cognitive assessment: (**a**) Corsi block task, where the textual prompts refer to “The number of mice appearing will be 2,” “Please remember,” “Please wait,” and “Please recall”; (**b**) Backward digit span task, where the textual prompts refer to “The number of digits heard will be 2,” “Please remember,” “Please wait,” and “Please recall”; (**c**) Numerical Stroop task; and (**d**) Symbolic approximate arithmetic task, where the textual prompts refer to “Left” and “Right”.

We measured visual–spatial WM using a modified Corsi block task for younger populations (Figure 1a; Pagulayan et al., 2006), which has demonstrated good reliability and validity in children (H. Liu et al., 2023). Cartoon mice appeared one after another within a 5 × 5 square grid to serve as stimuli. Following a 5000 ms retention interval, children were instructed to reproduce the spatial sequence by clicking the boxes in the exact order of presentation. To ensure full comprehension of the rules, a four-trial practice block was provided prior to the formal assessment, comprising two trials with a sequence length of two items and two trials with a length of four items. The experimental phase began with a two-item sequence and utilized a dynamic adjustment algorithm. Success on two straight trials at the same level triggered a one-item increase in sequence length. Conversely, the task ended if a child failed two straight trials at any difficulty level. The final visual–spatial WM metric represented the longest sequence length achieved across two successful consecutive trials as established by Vernucci et al. (2023).

#### 2.2.2. Backward Digit Span Task

We evaluated verbal WM through the backward digit span task as shown in Figure 1b (Waters & Caplan, 2003). Its sensitivity and reliability for younger populations are well documented in previous research (H. Liu et al., 2025). During the assessment, randomized digits ranging from 1 to 9 were presented aurally at a rate of one digit per second. Children submitted the digits into the answer windows using a backward arrangement. To ensure comprehension of the instructions, a practice phase consisting of four trials was conducted before the formal test commenced with a sequence length of two. Difficulty then increased using the same adaptive logic applied to the Corsi block task. According to the scoring method of Jones et al. (2022), the verbal WM score represented the longest sequence length correctly recalled in reverse over two consecutive trials.

#### 2.2.3. Numerical Stroop Task

The numerical Stroop task, illustrated in Figure 1c evaluated IC (Butterworth, 2003) and provides a reliable metric for distinguishing various levels of IC among school-age children (Berger et al., 2025; Farhi et al., 2024). Each trial commenced with a central fixation cross displayed for 250 ms, followed by a pair of digits ranging from 2 to 9 presented on a black background. Participants were instructed to identify the numerically larger digit while ignoring its physical size. The experiment included three conditions: (a) congruent, where the numerically larger digit was also physically larger (e.g., a large 9 at 128 × 200 pixels paired with a small 4 at 51 × 80 pixels); (b) incongruent, where the numerically larger digit was physically smaller (e.g., a small 9 at 51 × 80 pixels paired with a large 4 at 128 × 200 pixels); and (c) neutral, where digits differed in numerical value but were identical in physical size (64 × 100 pixels). The formal test consisted of 159 trials, with 53 trials per condition presented in a randomized order. Digit positions were counterbalanced across trials, and stimuli remained on the screen until a response was made. The indicator of IC was the mean reaction time for correct trials (Augustinova et al., 2022).

#### 2.2.4. Customized Arithmetic Task

EC ability was evaluated using a customized arithmetic task consisting of 38 items (Cui et al., 2019; L. Li et al., 2022). The task included addition, subtraction, and multiplication problems randomly assigned based on the participants’ current academic level. Specifically, simple multiplication items involved a two-digit multiple of 10 and another two-digit number (e.g., 30 × 36 = _), while addition and subtraction items comprised a combination of three-digit and two-digit numbers (e.g., 715 + 42 = _). Participants were instructed to complete the task within a 10 min time limit. Each correct response was awarded one point, while incorrect or unanswered items received zero points. The primary outcome measure was the total composite score. In the current sample, the task demonstrated good internal consistency, with Cronbach’s *α* of 0.96 for the pretest and 0.98 for the posttest.

#### 2.2.5. Symbolic Approximate Arithmetic Task

Children’s AC skills were assessed via a symbolic approximate arithmetic task (Figure 1d; Gilmore et al., 2007; Gunderson & Hildebrand, 2021). In each trial, two cartoon characters—a pig and a monkey—were presented, each possessing bags of candies with specific quantities labeled on the bags. For the pig on the left, an operation was performed indicating either the acquisition (+) or loss (−) of a second candy bag, while the monkey on the right held a single bag. Participants were required to estimate and determine which animal possessed a greater total number of candies. They were instructed to respond as quickly and accurately as possible within a 3 min time limit. The assessment comprised 30 trials, with addition and subtraction problems equally represented and presented in a counterbalanced sequence. Correct responses were awarded 1 point, whereas incorrect responses received 0 points. The final composite score, representing the total number of correct estimations, served as the primary indicator for this task. In the current sample, the task demonstrated good internal consistency, with Cronbach’s *α* of 0.95 for the pretest and 0.90 for the posttest.

#### 2.2.6. Integrated Adaptive Working Memory Training

Participants in the WM training group received a specialized IA-WMT program, delivered via a gamified mobile application (“AI Learning Ability Training” version 1.3.6; Miaoxiaohe Technology Co., Ltd., Shenzhen, China). To ensure high ecological validity and sustained engagement, the training was embedded within an ancient Chinese thematic map where participants completed tasks involving traditional dishes and numerical sequences. This IA-WMT program holistically integrated core cognitive mechanisms adapted from established paradigms—including the adaptive Corsi block, digit span and Stroop training tasks (Enge et al., 2014; Henry et al., 2022; Miura et al., 2017; Zhao & Jia, 2019)—into a unified training experience that addressed the updating, inhibition and shifting of the WM central executive (see Table 1). Each daily session lasted 40 min, consisting of four five-minute modules designed to tax the WM system under varying cognitive demands.

**Figure 2:**
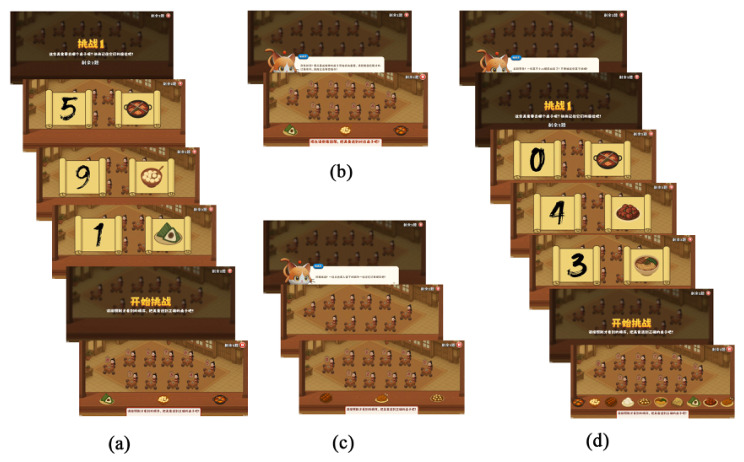
Examples of integrated adaptive working memory training tasks. Note. The panels display representative screenshots from the four training modules: (**a**) Sequential Sprint, where the textual prompts translate to “Challenge 1: Which table are these delicacies going to? Quickly remember their seats! 2 trials remaining,” “Start Challenge: Please deliver the delicacies to the correct tables in the order you just saw!” and “Please deliver the delicacies to the correct tables in the order you just saw!”; (**b**) Reverse Exploration, where the textual prompts translate to “Emergency! Food preparation must start in reverse order from the last table served. Please recall the order sequence backward as the kitchen is waiting for your instructions!” and “Now please recall backward and deliver the delicacies to the corresponding tables!”; (**c**) Sync Challenge, where the textual prompts translate to “Ultimate Challenge! Click on the copper coins left by guests while memorizing the order sequence!” and “Please deliver the delicacies to the correct tables in the order you just saw!”; and (**d**) Focus Breakthrough, where the textual prompts translate to “Kitchen Warning! Some dishes accidentally came out early! Do not be distracted by these dishes!” “Challenge 1: Which table are these delicacies going to? Quickly remember their seats!” “Start Challenge: Please deliver the delicacies to the correct tables in the order you just saw!” and “Please deliver the delicacies to the correct tables in the order you just saw!”.

The training experience progressed through four interconnected modules that utilized the same visual–auditory stimuli (dishes and numbers). In the initial phase, Sequential Sprint, children performed a forward-recall task where they matched dishes to numbered tables in the exact order of presentation (Figure 2a). This was followed by the Reverse Exploration phase, which taxed the mental manipulation of verbal and spatial information through backward-order matching (Figure 2b; Baddeley, 2012). The third module, Sync Challenge, required participants to execute preparatory responses by clicking randomly distributed copper coins before the memory stimuli would appear (Figure 2c). Finally, the Focus Breakthrough module challenged WM updating by introducing task-irrelevant distractor dishes during the retrieval stage, requiring participants to maintain target items while effectively managing interference (Figure 2d; Berger et al., 2025). Throughout all modules, each response was immediately met with distinctive auditory feedback consisting of specific sound effects indicating accuracy to facilitate reinforcement learning. Crucially, following an incorrect response, the system provided strategy-contingent instruction, such as mnemonic chunking to group multiple digits into single units.

The difficulty level was governed by a multidimensional adaptive algorithm that adjusted both the sequence length and the stimulus presentation duration. For sequence length, each trial consisted of two items at a given difficulty level. The assessment always commenced with a sequence length of one. Following the adaptive logic established in previous intervention literature, two consecutive correct trials resulted in a one-item increase in sequence length, whereas one error maintained the current level, and two consecutive errors led to a one-item reduction (Henry et al., 2014; Lau et al., 2025). Crucially, the stimulus presentation duration was also dynamically adjusted to maintain an optimal cognitive challenge: while the task initially commenced with a duration of 1000 ms per stimulus, accurate performance or improved speed led to a 50 ms decrease in subsequent display times. Errors or slower responses caused the timing to increase by 50 ms instead. This procedure ensured that the task remained at the edge of each participant’s cognitive capacity. To ensure continuity, the maximum difficulty level achieved in a session was automatically recorded and served as the starting baseline for the following day.

To quantify training progression, the system derived two primary indices: Memory Capacity (MC) and Memory Ability (*MA*). MC served as a raw behavioral indicator, defined as the peak sequence length achieved across the four modules within a single session. In contrast, *MA* was a composite proficiency score calculated via a non-linear algorithm that weighted task length (*L*) against the module-specific difficulty coefficient (*K*) and accuracy (*A*):

(1) $$MA\;=\;\frac{\sum_{j=1}^{n}\left(L_{j\;}\times \;K_{j}\;\times \;A_{j}\right)}{n}\;\times \;\omega$$

In this formula, K represents the difficulty weight assigned to each module (Sequential: 1.0; Reverse: 1.2; Focus: 1.3; Sync: 1.5), n is the total number of trials, *A* is the trial-level accuracy, and ω is a normalization constant. Compared to the raw MC, the *MA* provides a more nuanced reflection of information processing efficiency under varying cognitive loads. At the conclusion of each session, the terminal *L* parameters were recorded as the starting baseline for the subsequent day.

#### 2.2.7. Huarongdao Sliding Block Puzzle Training

Participants in the active control group engaged in sliding block puzzles via the “Huarongdao” module within a comprehensive mobile puzzle application (Sudoku 8.7; Wuhan Dobest Information Technology Co., Ltd., Wuhan, China). This specific module was selected to provide a cognitive and logical challenge that was developmentally appropriate for the participants’ age. Daily training sessions were fixed at 40 min, with the difficulty increasing gradually as participants completed successive levels. To ensure the non-cumulative and standardized nature of the intervention, each session was reset to the simplest difficulty level at the start of each day. During the task, participants were required to navigate sliding blocks within a restricted grid, utilizing logical deduction and spatial planning to achieve specific target spatial configurations.

### 2.3. Procedure

**Figure 3:**
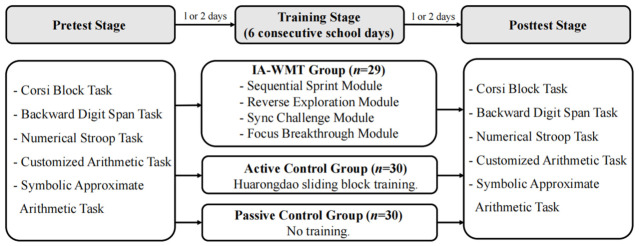
Flow diagram of the study procedure.

The experimental procedure consisted of three primary phases: a pretest assessment, a six-day intervention period, and a posttest assessment (Figure 3). The pretest and posttest sessions each lasted approximately 45 min and involved five distinct tasks. WM was evaluated using the Corsi block and backward digit span tasks, while IC was measured via the numerical Stroop task. These three assessments were programmed and administered using E-Prime 2.0 software (Psychology Software Tools, Inc., Sharpsburg, PA, USA) to ensure precise stimulus presentation and response recording. To evaluate transfer effects, a customized arithmetic task and a symbolic approximate arithmetic task were utilized. Although these arithmetic measures were sourced from the psychological assessment platform (http://www.dweipsy.com/lattice; accessed on 25 December 2024; Cui et al., 2019; Zhang et al., 2024), they were administered in a standardized paper-and-pencil format.

The intervention phase consisted of an intensive schedule of six sessions conducted over six consecutive school days. This short-term, high-intensity protocol was strategically designed based on a clear scientific rationale. First, in terms of neuroplasticity, evidence has confirmed that significant cognitive and neural modulations can be induced within remarkably brief windows. For instance, Tang et al. (2007) observed marked improvements in attention and self-regulation after only five days of intensive practice (20 min per day). Second, a comprehensive meta-analysis of children’s executive function training revealed that the total number of sessions is not a significant moderator of transfer effects, suggesting that increasing the duration does not necessarily yield superior gains (Kassai et al., 2019). Furthermore, the six-day duration was selected to accommodate the specific psychological characteristics of young children. As noted by Wass et al. (2012), maintaining motivation and engagement is critical when training young participants, as they often lack the metacognitive awareness required to voluntarily sustain effort. Consequently, research with younger populations frequently employs smaller training doses to ensure peak engagement. By concentrating the training into 6 consecutive sessions, we aimed to maximize the immediate induction of cognitive plasticity and adhered to the ecological constraints of the school’s weekly curriculum. Each training session lasted approximately 40 min. While the passive control group followed the standard school curriculum without additional intervention, participants in the IA-WMT and active control groups completed their respective programs on tablets provided by the research team. Posttest assessments were completed within one to two days following the final training session to capture the immediate effects of the intervention. All testing and training sessions were conducted in a dedicated computer classroom at the end of the school day, supervised by two to three trained experimenters who ensured task compliance and environmental consistency. Furthermore, the researchers administering the assessments remained strictly blinded to the participants’ group allocations during all testing phases.

### 2.4. Statistical Analyses

Statistical analyses were performed using SPSS 26.0 (IBM Corp., Armonk, NY, USA). Prior to the primary analyses, the data were screened for missingness. While no missing values were present during the assessment stages, a minor missing rate of 2.30% was observed for MC and *MA* during the training phase. The expectation-maximization (EM) algorithm was employed to handle these missing values (Dong & Peng, 2013). To evaluate the experimental effects, three steps of analyses were conducted. First, a multivariate analysis of variance (*MA*NOVA) was performed to test the homogeneity of baseline levels across groups, with group as the independent variable and pretest performance on each task as the dependent variables. The second stage of analysis focused on longitudinal progress during training through one-way repeated-measures ANOVAs with session as the independent variable. We also implemented linear regressions to model improvement trends by using session number as the predictor for training performance. Third, to evaluate the training and transfer effects of IA-WMT, a series of 3 (Group: IA-WMT, active and passive control) × 2 (Time: pretest, posttest) mixed-design ANOVAs or analyses of covariance (ANCOVAs) were conducted. Specifically, for dependent variables that significantly correlated with demographic factors, age and/or gender were entered as covariates. Dependent variables included spans from the Corsi and backward digit tasks, mean reaction times of the numerical Stroop task, and total scores of the customized arithmetic task and the symbolic approximate arithmetic task. Significant interactions led to simple effect probing with Bonferroni adjustments. No post hoc comparisons for main effects were necessary in the final reporting, as significant main effects either involved only two levels (i.e., Time) or were qualified by higher-order interactions. All statistical inferences relied on a *p* < .05 threshold and partial eta-squared ($\eta_{p}^{2})$ indicated the magnitude of each effect.

## 3. Results

### 3.1. Preliminary Analyses

Table 2 presents the means and standard deviations for all groups across pretest and posttest, along with the results of the correlational analyses. A multivariate analysis of variance (*MA*NOVA) was conducted using pretest performance on each task as dependent variables and results demonstrated that all tested cognitive abilities were statistically indistinguishable between groups at the pretraining stage, *F*(10, 164) = 0.73, *p* = .70. No significant group effects emerged for visual–spatial WM, *F*(2, 86) = 0.53, *p* = .59, verbal WM, *F*(2, 86) = 0.92, *p* = .40, IC, *F*(2, 86) = 1.15, *p* = .32, EC, *F*(2, 86) = 0.37, *p* = .69, AC, *F*(2, 86) = 0.52, *p* = .60. These findings verify that the randomization process successfully created balanced groups before the training protocol began.

### 3.2. Progress During Training Sessions

**Figure 4:**
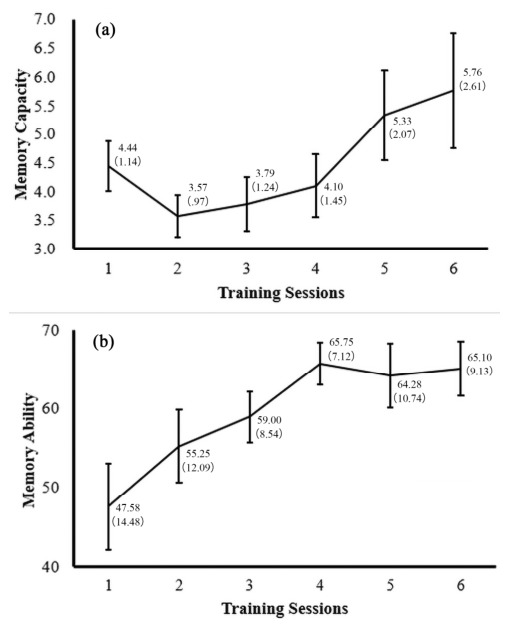
Developmental trajectories and descriptive statistics during training: (**a**) changes in Memory Capacity across six training sessions; (**b**) changes in Memory Ability across six training sessions. Note. Error bars represent 95% confidence intervals (CIs). The embedded data table presents the corresponding means, with *SD*s in parentheses.

Figure 4 presents the descriptive data for each task across the six intervention sessions for the IA-WMT group. Figure 4a illustrates the changes in MC across the six training sessions. To evaluate these changes, we utilized a one-way repeated-measures ANOVA with training session serving as the primary factor and MC as the dependent factor. Results revealed a significant main effect of session, *F*(5, 140) = 11.42, *p* < .001, $\eta_{p}^{2}$ = 0.29. Post hoc comparisons indicated a significant increase between Sessions 4 and 5 and a significant decrease between Sessions 1 and 2, while performance changes between all other consecutive sessions did not reach statistical significance. Notably, children’s performance in the 6th session was significantly higher than in the first session (*MD* = 1.32, *p* < .05).

Figure 4b illustrates the changes in *MA* across the six training sessions. A similar model was applied to *MA* and identified a significant main effect for session, *F*(5, 140) = 17.84, *p* < .001, $\eta_{p}^{2}$ = 0.39. Post hoc comparisons revealed significant improvements from Session 1 to 2 and Session 3 to 4, whereas no significant changes were observed between other consecutive sessions. Performance in the sixth session was significantly superior to that in the 1st session (*MD* = 17.52, *p* < .001).

Furthermore, linear regression analyses were conducted with session as the predictor and training performance as the outcome. The regression analysis confirmed that session significantly predicted the advancement in both MC (*R*^2^ = 0.11, *p* < .001) and *MA* (*R*^2^ = 0.23, *p* < .001). Detailed regression parameters are displayed in Table 3. Collectively, these findings suggest that children’s WM performance improved progressively throughout the training period.

### 3.3. Effectiveness of IA-WMT

#### 3.3.1. Visual–Spatial Working Memory

**Figure 5:**
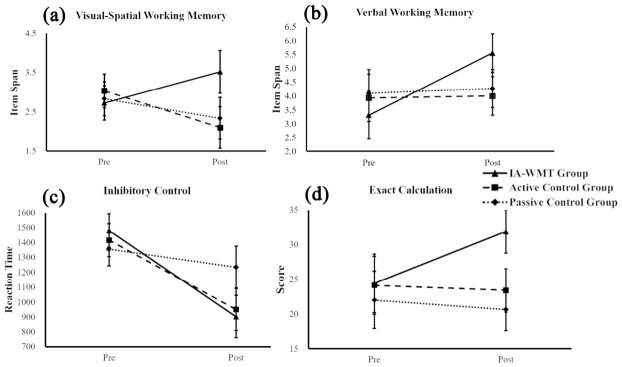
Changes in pretest and posttest performance of each group across the cognitive and academic tasks. Note. The panels illustrate the performance trajectories for each specific domain: (**a**) visual–spatial working memory; (**b**) verbal working memory; (**c**) inhibitory control, where a decrease in reaction time signifies an advancement in performance; and (**d**) exact calculation. The lines with error bars represent the means and 95% CIs of each group at pretest and posttest.

Results revealed a significant Time × Group interaction, *F*(2, 86) = 8.08, *p* < .001, $\eta_{p}^{2}$ = 0.16. However, the main effects of Group, *F*(2, 86) = 2.82, *p* = .07, and Time, *F*(1, 86) = 1.38, *p* = .24 were not significant. As illustrated in Figure 5a, the IA-WMT group showed a significant increase in item span from pretest to posttest (*MD* = 0.79, *p* < .05), whereas the active control group exhibited a significant decrease (*MD* = −0.93, *p* < .01); no significant change was observed in the passive control group (*MD* = −0.50, *p* = .11). Furthermore, the simple effect of group at posttest indicated that the IA-WMT group significantly outperformed both the active control group (*MD* = 1.42, *p* < .01) and the passive control group (*MD* = 1.18, *p* < .05), while no significant difference was found between the active and passive control groups (*MD* = −0.23, *p* = 1.00). These findings suggest that IA-WMT, rather than Huarongdao training, effectively enhanced children’s visual–spatial WM.

#### 3.3.2. Verbal Working Memory

Results revealed a significant main effect of Time, F(1, 86) = 9.41, p < .01, $\eta_{p}^{2}$ = 0.10, and a significant Time × Group interaction, *F*(2,86) = 6.87, *p* < .01, $\eta_{p}^{2}$ = 0.14. The main effect of Group was not significant, *F*(2,86) = 0.54, *p* = .59. As illustrated in Figure 5b, simple effect analyses indicated that the IA-WMT group demonstrated a significant increase in digit span from pretest to posttest (*MD* = 2.24, *p* < .001), whereas no significant changes were observed in the active control group (*MD* = 0.07, *p* = .89) or the passive control group (*MD* = 0.17, *p* = .72). At posttest, the IA-WMT group significantly outperformed both the active control group (*MD* = 1.55, *p* < .01) and the passive control group (*MD* = 1.29, *p* < .05), while no significant difference was found between the active and passive control groups (*MD* = −0.27, *p* = 1.00). These findings suggest that IA-WMT, rather than Huarongdao training, led to significant improvements in children’s verbal WM, as indexed by backward digit span performance.

#### 3.3.3. Inhibitory Control

The mean reaction times of the numerical Stroop task served as the dependent variable for a 3 (Group: IA-WMT, active and passive control) × 2 (Time: pretest, posttest) mixed-design ANCOVA, with age and gender entered as covariates. Results revealed non-significant main effects of Time, F(1, 84) = 2.50, p = .12, and Group, F(2,84) = 1.97, p = .15, but a significant Time × Group interaction, F(2,84) = 6.13, p < .01, $\eta_{p}^{2}\;$= 0.13. As illustrated in Figure 5c, simple effect analyses indicated that both the IA-WMT group (*MD* = −576.85, *p* < .001) and the active control group (*MD* = −454.08, *p* < .001) demonstrated a significant decrease in reaction times from pretest to posttest, whereas no significant changes were observed in the passive control group (*MD* = −132.00, *p* = .16). At posttest, both the IA-WMT group (*MD* = −327.39, *p* < .01) and the active control group (*MD* = −270.74, *p* < .05) significantly outperformed the passive control group, while no significant difference was found between the IA-WMT and active control groups (*MD* = −56.65, *p* = 1.00). These findings suggest that both IA-WMT and Huarongdao training led to significant improvements in children’s IC, as indexed by reduced reaction times in the numerical Stroop task.

#### 3.3.4. Exact Calculation

The scores of the customized arithmetic task served as the dependent variable for a 3 (Group: IA-WMT, active and passive control) × 2 (Time: pretest, posttest) mixed-design ANCOVA, with age entered as a covariate. Results revealed a non-significant main effect of Time, *F*(1, 85) = 0.81, *p* = .37, but a significant main effect of Group, *F*(2, 85) = 5.04, *p* < .01, $\eta_{p}^{2}\;$= 0.11. Crucially, a significant Time × Group interaction was observed, F(2, 85) = 5.44, p < .01, $\eta_{p}^{2}\;$= 0.11. As illustrated in Figure 5d, simple effect analyses indicated that the IA-WMT group demonstrated a significant increase in EC performance from pretest to posttest (*MD* = 7.48, *p* < .001), whereas no significant changes were observed in either the active control group (*MD* = −0.73, *p* = .72) or the passive control group (*MD* = −1.37, *p* = .51). At posttest, the IA-WMT group significantly outperformed the active control group (*MD* = 8.91, *p* < .001) and the passive control group (*MD* = 11.05, *p* < .001). No significant distinction was found between the active and passive control groups (*MD* = 2.14, *p* = 1.00). These findings suggest that IA-WMT, rather than Huarongdao training, led to significant transfer effects on children’s EC ability.

#### 3.3.5. Approximate Calculation

The scores of the symbolic approximate arithmetic task served as the dependent variable for a 3 (Group: IA-WMT, active and passive control) × 2 (Time: pretest, posttest) mixed-design ANCOVA, with gender entered as a covariate. Results revealed a significant main effect of Time, F(1, 85) = 95.81, p < .001, $\eta_{p}^{2}\;$= 0.53, with an overall improvement in performance across sessions (*MD* = 11.76). However, the main effect of Group was non-significant, *F*(2, 85) = 2.55, *p* = .08. Crucially, the Time × Group interaction also failed to reach significance, *F*(2, 85) = 2.23, *p* = .11, suggesting that the rate of improvement did not differ significantly among the three groups. These findings indicate a general practice effect on the symbolic approximate arithmetic task, with neither IA-WMT nor Huarongdao training producing specific transfer gains beyond this effect.

## 4. Discussion

The present study indicates that six sessions of IA-WMT yield robust training effects on WM and suggests potential asymmetric transfer to IC and EC, though no specific gains were observed for AC. By deconstructing mathematical abilities from a dual-system perspective, these findings help outline the cognitive boundaries where plasticity translates into academic gains during the school years. Collectively, the results suggest that the synergistic integration of executive components provides a promising framework for cognitive enhancement while highlighting the asymmetric nature of transfer based on the inherent demands of the academic task.

### 4.1. Synergistic Design of the Integrated Paradigm Dictates the Robust Effectiveness of Working Memory Training in School-Age Children

Children in the IA-WMT group exhibited significant improvements in both MC and *MA* following the six-session protocol. These results are consistent with meta-analytic evidence suggesting that adaptive WMT effectively enhances the short-term performance of WM in school-age children (Kassai et al., 2019; Melby-Lervåg et al., 2016; Sala & Gobet, 2017). Notably, the observed decline in MC from Session 1 to Session 2 requires careful interpretation. Rather than indicating cognitive regression, this dip likely represents an initial adaptation phase where the algorithm corrects for potential novelty-driven over-performance in the first session (Ricci et al., 2024; Tatalovic, 2009). Because the IA-WMT protocol adopts the peak performance of the previous day as the starting baseline, the novelty-induced peaks in session 1 led to an excessively high difficulty level at the beginning of session 2. Under these circumstances, children who had not yet fully adapted to such high-load demands exhibited impaired performance within the session (Gutierrez et al., 2021; Herz & Zihlmann, 2024; Ren et al., 2025). Crucially, the discrepancy between the transient decline in MC and the improvement of *MA* highlights the differential sensitivity of the assessment indices. As a raw peak-load metric, MC is susceptible to the stochastic nature of threshold testing. In contrast, the composite *MA* index provides a more nuanced and stable reflection of information processing efficiency by weighting sequence length against task difficulty and trial-level accuracy. The fact that *MA* did not exhibit a parallel decline suggests that while children’s peak capacity fluctuated during the calibration phase, their underlying executive coordination remained robust and began to optimize early in the training process.

The results revealed by the composite *MA* scores indicated that no significant gains occurred after the fifth session compared to the fourth session, with performance remaining stable thereafter, which aligns with findings from previous meta-analyses regarding the diminishing returns of excessive training frequency in typically developing children (Kassai et al., 2019; Scionti et al., 2020). This plateau suggests that for typically developing samples during the school years, total training duration may be a more critical predictor of enhancement than the absolute frequency of sessions, provided that motivation is sustained and fatigue is mitigated. Unexpectedly, the decline in visual–spatial WM within the active control group may stem from previously acquired cognitive routines affecting subsequent task performance (Ni et al., 2023; Reimer et al., 2002). Nevertheless, this interpretation remains tentative. Alternative explanations cannot be entirely ruled out, and future research is therefore required to elucidate the precise mechanisms underlying this specific outcome. The specific spatial routines formed during the Huarongdao task might have been applied to the Corsi block task even though they were inappropriate. This phenomenon reflects an asymmetric negative transfer effect, characterized as a strong-but-wrong sequence application that manifests as negative transfer errors on untrained static tasks (Ni et al., 2023; Woltz et al., 2000). The robust effectiveness of IA-WMT is primarily attributable to the synergistic advantages of the integrated paradigm, which targets the central executive by simultaneously activating multiple sub-components such as updating, inhibition, and shifting (Baddeley, 2012; Miyake et al., 2000). Unlike traditional single-task drills that often foster task-specific strategies rather than broad cognitive reorganization, the holistic design of IA-WMT requires children to continuously engage central executive coordination to manage diverse task demands (Basak et al., 2020; Shipstead et al., 2012). In addition to expanding core capacity, the intervention provides explicit mnemonic guidance, such as chunking, to equip children with the cognitive tools necessary for efficient information processing (Assecondi et al., 2021; Ben Izhak & Lavidor, 2023). The structural integrity of this framework is further fortified by the synergy between gamification and adaptive mechanisms, which ensures sufficient training dosage and optimal neuroplasticity (Shaheen et al., 2025; Sweller, 2020). While high-intensity protocols often induce cognitive fatigue, the scenarios and gamified elements served as structural scaffolds to alleviate fatigue and bolster intrinsic motivation (Cicerone et al., 2019; Czeropski & Pembrook, 2015; Wang et al., 2025). Crucially, the adaptive algorithm maintains task difficulty at each individual’s edge of ability, which may serve as a driver for neuroplasticity by providing the necessary drive for functional reorganization within the executive system (Delalande et al., 2020; Jones et al., 2022; Sweller, 2020).

### 4.2. Enhanced Executive Coordination Facilitates near Transfer to Inhibitory Control Through Proactive Strategy Shifts

The results indicated that IA-WMT effectively induced significant near transfer to IC, as demonstrated by the marked reduction in reaction times during the numerical Stroop task. For the IA-WMT group, this improvement aligns with the theoretical proposition that IC functions as a cognitive gatekeeper whose efficiency is fundamentally linked to the overall capacity and coordination of the central executive (Chiew & Braver, 2017; Dempster, 1992). By embedding inhibitory demands into updating paradigms within the IA-WMT framework, the intervention likely optimized the underlying executive attention efficiency required to maintain goal representations while filtering task-irrelevant distractions. As children expand their working memory capacity, they gain sufficient cognitive resources to transition from late-stage reactive corrections to pre-emptive proactive control strategies (Berkman et al., 2014; Munakata et al., 2011; Troller-Renfree et al., 2020). Crucially, while previous research has frequently reported null or highly restricted near-transfer effects to IC following conventional WMT (Kassai et al., 2019; Melby-Lervåg et al., 2016), recent empirical studies demonstrate that WMT can successfully transfer to IC in school-age children when interventions utilize intensive or adaptive designs (Berger et al., 2025; H. Liu et al., 2023). This integrated approach drives the profound functional reorganization necessary for genuine transfer, further highlighting the necessity of utilizing sensitive reaction-time metrics to capture plasticity in populations prone to accuracy ceiling effects (Diamond & Ling, 2016; Willoughby et al., 2023).

Notably, the active control group also exhibited significant reductions in IC reaction times, which necessitates a detailed examination of the underlying cognitive mechanisms within the Huarongdao task. Sharing a structural cognitive isomorphism with classic executive paradigms such as the Tower of London, this task requires participants to navigate objects within restricted spatial configurations to reach a target state (Baughman & Cooper, 2007; Newman et al., 2003). Successful performance on such tasks relies heavily on inhibitory processes to suppress impulsive moves that violate specific rules (Welsh et al., 1999; Zook et al., 2004). Consequently, the frequent requirement to inhibit suboptimal paths likely activated and reinforced the same cognitive control resources that support behavioral inhibition in the Stroop task. Additionally, high-intensity cognitive engagement can induce a state of heightened phasic alertness, which significantly increases visual processing speed and executive alertness (Benzing et al., 2016; Petersen et al., 2017). This state of elevated mental investment likely facilitated a general enhancement in processing efficiency across the active control group. Overall, these findings provide important preliminary evidence regarding the malleability of IC. By acknowledging the distinct mechanisms driving these improvements, this study suggests that high-intensity cognitive interventions are viable and essential methods for bolstering IC, whether achieved through the transfer effects of organically integrated WM paradigms or the direct inhibitory demands of complex spatial logic puzzles.

### 4.3. Differential Cognitive Resource Dependency Dictates the Boundary of Far Transfer to Mathematical Abilities

The findings revealed a significant functional dissociation in far-transfer effects, in which IA-WMT significantly enhanced EC performance while yielding no specific gains for AC. This asymmetrical improvement provides promising behavioral alignment with the dual-system model of mathematical cognition (Dehaene et al., 1999; Stanescu-Cosson et al., 2000; Yue et al., 2025). Critically, this study extends previous work by suggesting that training-induced WM expansion may primarily benefit those mathematical operations that explicitly recruit executive resources. Rather than merely reiterating that EC relies on the central executive for algorithmic retrieval, the current data empirically highlight this dependency. The magnitude of EC improvement was substantial. With a mean increase of 7.48 points, this gain accounts for approximately 20% of the total task score and represents an effect size of 0.65 standard deviations. This increase is comparable to or noticeably larger than those reported in recent meta-analyses regarding WM training on mathematics (Aksayli et al., 2019; Sala & Gobet, 2020), suggesting that the integrated IA-WMT paradigm is particularly effective for academically relevant outcomes when there is a strict functional overlap between the trained executive processes and the inherent task demands. Even so, it cannot be entirely ruled out that EC performance was indirectly enhanced through alternative pathways, such as improved attention or enhanced test-taking skills.

Conversely, the lack of specific transfer to AC highlights the potential boundaries of cognitive plasticity when applied across distinct cognitive domains. This difference can be elucidated through the distinct cognitive demands of the two tasks. AC primarily activates the approximate number system, which operates relatively independently of precise WM resources (Kucian et al., 2008; Stanescu-Cosson et al., 2000). Consequently, the expansion of executive capacity induced by IA-WMT did not catalyze specific gains in intuitive estimation. While children demonstrated overall improvements in estimation, likely due to general practice or maturation, these gains did not differ significantly across groups (Boot et al., 2013; Wilkey et al., 2022). By identifying the task-specific cognitive structure as a critical boundary condition, these results offer a more nuanced resolution to the long-standing debate regarding whether WMT can effectively enhance mathematical abilities. This research suggests that the spillover effects of training are strictly constrained by the degree of functional overlap between the trained executive processes and academic demands. This evidence helps delineate these constraints of cognitive plasticity and suggests that WMT may operate as a targeted tool, rather than a universal instrument for academic enhancement during the school years.

### 4.4. Limitations and Future Work

The current research has achieved significant progress in validating the effectiveness of IA-WMT, although several limitations remain. First, as a preliminary exploration of this integrated adaptive training paradigm, the study focused on a sample of Chinese school-age children from a specific region. Future research should expand these efforts to diverse cultural backgrounds and varied populations to verify the generalizability of the findings. Second, the current experimental design primarily emphasizes the immediate effects of the intervention. Due to the absence of a delayed posttest, the study cannot assess whether the observed gains in IC and EC are maintained as stable cognitive traits once the intervention concludes. Future investigations must implement longitudinal tracking designs with delayed assessments to better explore how the early expansion of executive capacity predicts long-term academic growth trajectories during the school years. Third, because core features such as adaptivity, gamification, and explicit strategy instruction were bundled into a single protocol, the current design could not further explore which specific component drove the observed effects. Whether these elements operated independently or interacted synergistically remains unclear. These features represent critical mechanisms rather than irrelevant variables, and they deserve systematic evaluation in future research. Finally, because strategy self-reports were not included in the posttest, it remains unclear whether the transfer gains resulted from the pure expansion of WM capacity or from the internalization and application of more efficient problem-solving strategies (Fellman et al., 2020; Himi et al., 2023; Malinovitch et al., 2021).

Future research trajectories should strive to enhance the validity and reliability of cognitive training effects through more diverse and objective assessment methods. Integrating neuroimaging techniques such as functional magnetic resonance imaging or electroencephalography would help elucidate the neural mechanisms and cortical plasticity underlying this integrated paradigm. Research should also explore how initial cognitive profiles predict academic growth to provide a basis for the design of personalized intervention programs. Through these efforts, studies will more clearly delineate the boundary conditions of cognitive plasticity and offer cautious insights into how laboratory findings might eventually support practical classroom applications. Ultimately, these endeavors will further clarify these mechanisms and assist in the development of targeted interventions for diverse educational requirements.

## 5. Conclusions

The present study aimed to resolve the efficacy paradox in WMT by implementing an integrated adaptive paradigm for school-age children. The findings offer promising evidence that targeting the central executive of WM holistically yields immediate cognitive reorganization. Theoretically, this research provides vital empirical support for the dual-system model of mathematical cognition. By demonstrating that the integrated training selectively enhances EC while leaving AC unaffected, the study suggests that cognitive plasticity is not a generalized spillover effect. Instead, far transfer appears to be strictly governed by the degree of functional overlap between the trained executive processes and the inherent demands of the academic task. This asymmetric pattern helps outline the potential boundaries of cognitive malleability.

From an educational and practical perspective, these preliminary findings suggest the potential utility of moving beyond isolated cognitive drills in developmental interventions. The success of the integrated framework highlights the practical value of embedding high-intensity executive demands within gamified and ecologically valid scenarios. This synergistic approach ensures that children can sustain the necessary motivation and mitigate cognitive fatigue during critical developmental windows. Because IC and EC are foundational for behavioral regulation and academic achievement in formal schooling, this integrated training paradigm represents a promising and targeted intervention tool. Ultimately, this study indicates that when cognitive training is organically integrated and strategically deployed, it possesses the potential to optimize meaningful cognitive and academic development during the school years.

## Figures and Tables

**Table 1:** Specific central executive functions associated with each training module.

Module Name	Updating	Inhibition	Shifting
Sequential Sprint	**Strong**	Weak	Negligible
Reverse Exploration	**Very Strong**	Moderate	Weak
Sync Challenge	Moderate	Moderate	**Very Strong**
Focus Breakthrough	Strong	**Very Strong**	Weak

**Table 2:** Descriptive statistics of all variables at pretest and posttest.

Variables	1	2	3	4	5	6	7	8	9	10	11	12
**1. Gender**	1											
**2. Age**	0.15	1										
**3. Pre Visual−Spatial WM**	0.05	0.09	1									
**4. Post Visual−Spatial WM**	0.21	0.09	0.12	1								
**5. Pre Verbal WM**	−0.05	0.01	0.24 *	−0.15	1							
**6. Post Verbal WM**	−0.02	−0.12	0.11	0.43 ***	0.24 *	1						
**7. Pre IC**	0.33 **	0.23 *	0.20	0.25 *	−0.07	−0.08	1					
**8. Post IC**	−0.06	−0.14	0.12	−0.14	0.03	−0.10	−0.13	1				
**9. Pre EC**	0.05	0.22 *	0.13	0.27 **	0.18	0.24 *	−0.03	0.04	1			
**10. Post EC**	−0.11	0.14	−0.02	0.39 ***	−0.01	0.41 ***	0.08	−0.06	0.38 ***	1		
**11. Pre AC**	0.19	0.10	0.07	−0.07	0.12	−0.02	0.05	−0.04	0.13	0.00	1	
**12. Post AC**	0.25 *	0.08	0.09	0.24 *	0.18	0.30 **	0.17	−0.08	0.41 ***	0.44 ***	0.03	1
***M***	0.48	9.09	2.87	2.64	3.79	4.60	1416.45	1030.93	23.53	25.26	11.43	23.31
***SD***	0.50	0.32	1.17	1.58	2.35	2.00	322.15	408.88	11.52	9.68	8.22	5.54

Note. *M* = mean; *SD* = standard deviation; WM = working memory; IC = inhibitory control; EC = exact calculation; AC = approximate calculation. * *p* < .05, ** *p* < .01, *** *p* < .001.

**Table 3:** Performance in each session regressed to session in the IA-WMT group.

	*B*	*SE*	*β*	*t*	*p*	*R* ^2^
**Memory Capacity**						
**Session**	0.35	0.08	0.33	4.50	<.001	0.11
**Memory Ability**						
**Session**	3.47	0.48	0.48	7.21	<.001	0.23

## Data Availability

The data presented in this study are available on request from the corresponding author. The data are not publicly available due to privacy restrictions.

## Abbreviations

The following abbreviations are used in this manuscript:

WM	Working Memory
WMT	Working Memory Training
IA-WMT	Integrated Adaptive Working Memory Training
IC	Inhibitory Control
EC	Exact Calculation
AC	Approximate Calculation
